# The study of kinetic of silver catalytic degradation of phoxim

**DOI:** 10.1016/j.mex.2024.102927

**Published:** 2024-09-06

**Authors:** Gholamreza Shams, Mohammad Javad Chaichi, Jalal Hassan, Ali Pourshaban-Shahrestani

**Affiliations:** a Department of Chemistry, University of Mazandaran, Babolsar, Iran; b Department of Comparative Bioscience, Faculty of Veterinary Medicine, University of Tehran, Tehran, Iran

**Keywords:** Phoxim, Kinetic, Arrhenius plot, Degradation, Silver ion, HPLC, Green analysis

## Abstract

In this study, we scrutinized the degradation process of phoxim in the presence of Ag+ ions, maintaining a 1:1 molar ratio under diverse temperature conditions. Phoxim was chosen as the model compound to devise experimental methodologies that would shed light on the kinetic and degradation pathways within a time span of 0 to 184 min across varying temperatures. The Arrhenius equation was harnessed to ascertain the activation energies linked with the degradation of phoxim. The application of the Arrhenius equation enables the computation of the reaction constant at a given temperature, thereby paving the way for the prediction of phoxim concentrations at different temperatures. The second-order rate constant for the reaction was observed to lie within the range of 0.035 to 0.128 L mol-1min-1, and the half-life of the reaction fluctuated between 5.2 and 17 min across different temperatures.

• The study investigates the degradation of phoxim in the presence of Ag+ ions at various temperatures.
• The Arrhenius equation was used to calculate the activation energies and predict phoxim concentrations at different temperatures.
• The second-order rate constant for the reaction ranged from 0.035 to 0.128 L mol-1min-1, with the half-life varying between 5.2 and 17 min.

## Graphical abstract

**Figure:**
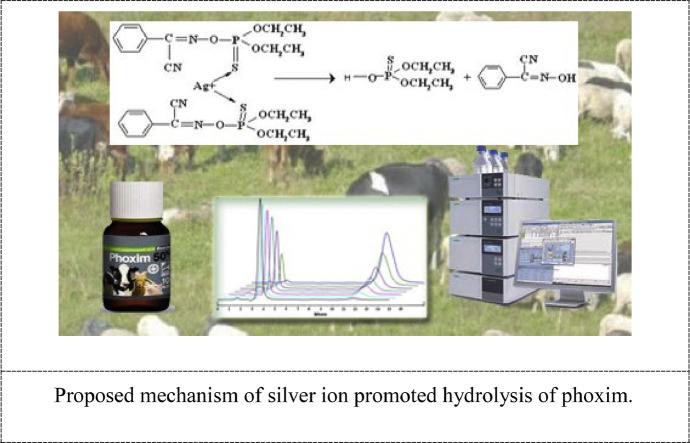

**Table:** 

Subject area:	Environmental Science
More specific subject area:	*Analysis*
Name of your method:	Green analysis
Name and reference of original method:	*EPA*
Resource availability:	*No*

## Background

Organophosphate pesticides (OPPs), known for their high acute toxicity, emerged as the primary substitutes for organochlorine pesticides globally for agricultural pest control in the 1960s and 1970s [1,2]. Phoxim, a member of the organophosphorus group, is a potent agent that diminishes blood cholinesterase levels upon ingestion or dermal exposure. Despite the oral LD50 toxicity level of phoxim in mice exceeding 2000 mg/kg, its widespread use as a spray or bath for ectoparasite eradication can lead to significant environmental contamination. The persistence of this pesticide in soil spans several months, posing a severe threat to aquatic life, avian species, and bees. Even when applied correctly in livestock management, phoxim can be detrimental to the environment. As with all organophosphate insecticides, phoxim acts on the nervous system of parasites (as well as mammals, birds, fish, and numerous other organisms) by inhibiting acetylcholinesterase (also known as AchE), an enzyme that releases and hydrolyzes acetylcholine (Ach). Ach plays a crucial role in transmitting nerve signals at neuromuscular junctions and between neurons in the brain (known as cholinergic brain synapses). Atropine, a parasympathetic drug, serves as an antidote for acute muscarinic symptoms. OPPs have been included in several priority pollutant lists in numerous countries due to their extensive use, high toxicity to biotic systems, and potential for discharge into aquatic environments. In recent years, several governmental agencies, including the USEPA, have begun to reevaluate the widespread use of organophosphates due to concerns about their impact on the human central nervous system, particularly in children. Given the established toxicity of OPPs to humans, their presence in the environment, including many of their degradation products found in surface water and groundwater, is of significant concern. Some OPP reduction processes may be inefficient or environmentally harmful due to the formation of mildly or acutely toxic by-products. Various methods of pesticide degradation have been explored, including oxidation [3,4], photodegradation [5], microbial decomposition [6] and hydrolysis [4]. Hydrolysis of OPPs can occur homogeneously, where H2O and OH– act as nucleophiles, and the rate of hydrolysis is catalyzed by the presence of metal ions. A critical aspect of phosphorus pesticide degradation is the identification and characterization of degradation products [[7], [8], [9]].

The environmental fate of organophosphorus pesticides is dictated by a multitude of factors, among which the chemistry of their aquatic milieu is of significant importance. In numerous instances, metal ions have been demonstrated to act as catalysts in the degradation of organophosphorus pesticides, with several hypotheses proposed to elucidate the precise mechanism of metal ion-mediated hydrolysis. In the context of this study, we have undertaken an investigation into the degradation of phoxim in the presence of silver ions across a range of temperatures. Given the established environmental impacts of phoxim, the objective of this research is to monitor the structural alterations it undergoes upon exposure to silver, with the ultimate aim of devising strategies for its environmental remediation. This represents a relatively unexplored area of research. Our findings indicate that at a Ag+ molar ratio exceeding 10, phoxim undergoes complete degradation. Furthermore, our study provides a basis for predicting and practically applying the degradation of phoxim at various temperatures.

## Method detail

### Reagent

Methanol, silver nitrate, deionized water, and phoxim were obtained from Merck (Germany) with analytical grade purity. Silver nitrate (Merck) solution (1.6 mol l^-1^) was prepared by dissolving the weighed amount of AgNO_3_ in 2.5 mL of methanol and 2.5 mL of water. Phoxim was prepared from Ehrnestorfer (Augsburg, Germany) and the stock solutions (1.6 mmol l^-1^) were prepared in methanol. Working standards of phoxim in the range of 0.16–1.6 mmol l^-1^ were made from stoke solution and were used for the calibration curve. All standards were stored in a refrigerator at 4 °C before use.

### Kinetic method

0.500 mL phoxim (3.2 mmol l^-1^) and 0.500 mL of Ag^+^ (3.2 mmol l^-1^) were mixed in autosampler vial to obtain Ag^+^ to phoxim mole ratio of 1:1 and kinetics was followed by HPLC on 0–184 min (with 17 min interval) at 25 °C. To study the kinetic constant at different temperatures, prepared sample was placed in the oven at the desired temperature and 20 microliters of it Immediately, was injected into the HPLC at the desired times. Each experiment was repeated three times.

### Instrumentation

High-performance liquid chromatography (Knauer, Germany) equipped with four pumps, degassing chamber, photodiode array detector, and an autosampler was used to measure the phoxim. Isolation was performed with a C_18_ column with dimensions of 30 cm in length and 4 mm in inner diameter and a particle size of 5 μm made by Waters (USA). EZ Chrom software was used to control the instrument, and areas of peak were obtained with integration at retention time of phoxim and its metabolite. The isocratic mobile phase of methanol and water with a ratio (50:50) at a flow rate of 1.0 mL min^-1^ and at a temperature of 25 °C (ambient temperature) was used and a detector wavelength of 282 nm was used for isolation and measurement. The volume of injection was 20 µL.

## Method validation

**Fig. 1:**
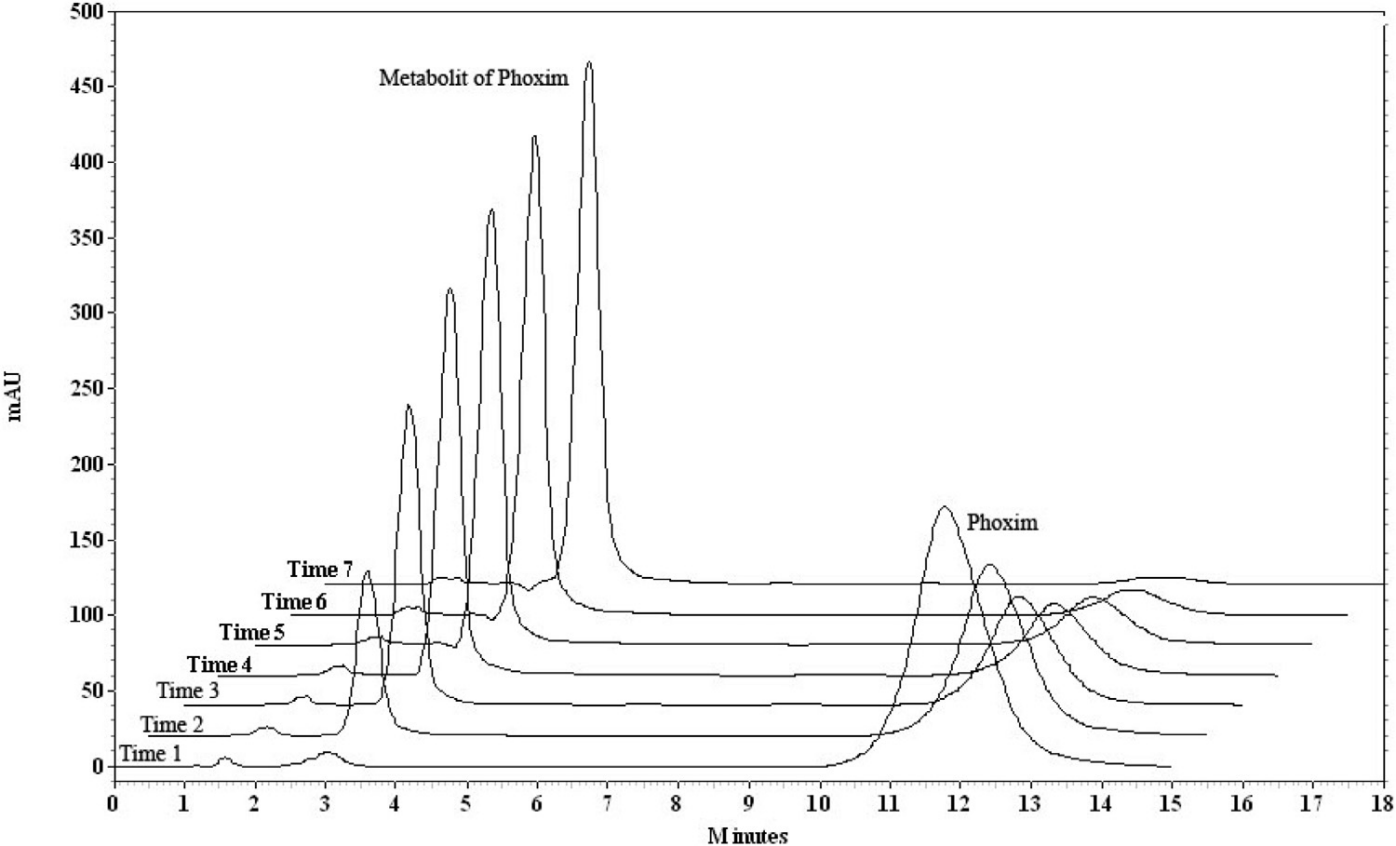
Chromatograms obtained for phoxim (1.6 mmol l^-1^) in the presence of silver nitrate (1.6 mmol l^-1^) at different times (0–184 min and 26 min interval).

Some primary studies and also our previous work investigate phoxim was degraded with Ag^+^ at silver ion/pesticide ratio ≤ stoichiometry and completely decomposed at a higher ratio. Thus, for kinetic investigation, phoxim to silver ion ratio of 1 was selected (Pehkonen and Zhang 2002). The chromatogram resulting from these changes can be seen in Fig. 1. In the obtained chromatograms, it is observed that overstudied time, due to the reaction of the phoxim with silver ion the amount of phoxim (retention time =12 min) decreases, and the amount of metabolite (retention time =2.95 min) increases.

## Results and discussion

### Degradation kinetics

#### Second-order reactions

The mathematical representation of the correlation between reactant concentrations and the rate of reaction is known as the rate law or velocity equation. In this equation, the rate constant ‘k’ is a crucial factor, which is contingent upon the temperature, the characteristics of the reactants, and the activation energy of the reaction. This constant is empirically determined for each distinct reaction.

(1) $$R=k{\left[A\right]}^{m}{\left[B\right]}^{n}$$

The exponents of concentrations (m and n) in the rate law can be either integer or fractional. The instantaneous rate of reaction can be deduced from the rate law. In a second-order reaction, the overall order is two, implying that the rate of reaction is proportional to the product of the concentrations of the reactants. The integrated second-order rate law is:

(2) $$\frac{1}{A_{t}}=kt+\frac{1}{A_{0}}$$

The exponents m and n are determined by the reaction order, which is dependent on the reaction type and mechanism. For elementary reactions, these exponents are equivalent to the stoichiometric coefficients of the reaction. However, for complex or multi-step reactions, the rate law must be accurately determined based on the reaction mechanism. In the context of the integrated second-order rate law, ‘A_t_’ signifies the concentration of the reactant of interest at a specific time, while ‘A_0_’ denotes the initial concentration. The second-order rate constant ‘k’ has units of L mol-1 time-1. A plot of $\frac{1}{A_{t}}$versus time ‘t’ yields a linear graph with a slope equal to ‘k’.

The half-life of a reaction, which is the time required for the concentration of the reactant to reduce by half, is a significant parameter. For a second-order reaction, the half-life is dependent on the initial concentration and can be calculated using a specific formula:

(3) $$t_{\frac{1}{2}}=\frac{1}{k{\left[A\right]}_{0}}$$

**Fig. 2:**
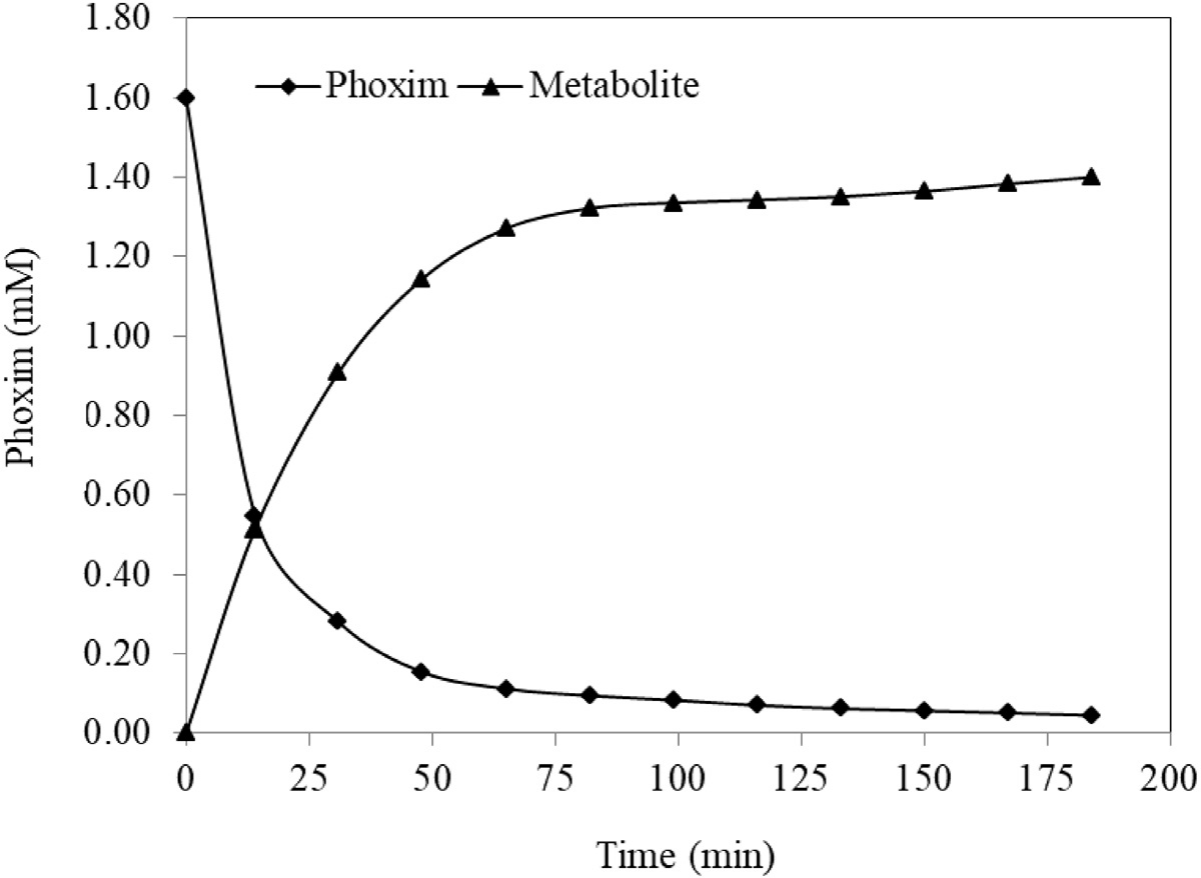
Concentration profile for phoxim and its metabolite against time.

The degradation of phoxim over time in the presence of Ag^+^ was monitored by using HPLC at different temperature. By plotting the concentration of phoxim in the presence of silver ions with a molar ratio of 1: 1 at different times, the Fig. 2 was obtained:

And by inverting plot of $\frac{1}{A_{t}}$vs. time (t) the following figure is obtained: which shows that it is proportional to the second power of phoxim and is of the second order, while in lower molar ratios of silver than phoxim (1:4), the reaction kinetics are first order. In other words, by doubling the concentration of phoxim, its degradation rate is increases four times in the presence of silver ions:

(4) $$R=k{\left[\text{phoxim}\right]}^{2}$$

**Scheme 1:**
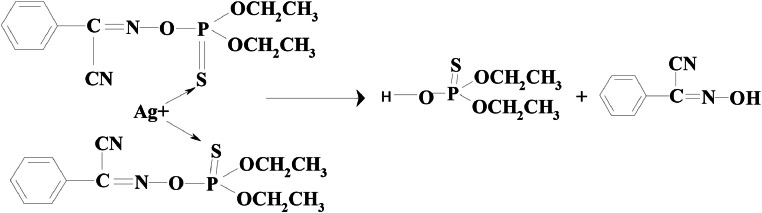
Proposed reaction mechanism of silver ion promoted hydrolysis of phoxim.

According to previous experiment proposed reaction mechanisms of phoxim is given as Scheme 1 [7,10].

Catalyst is a substance that can increase the rate of reaction while remaining unchanged during the reaction. Catalysts change the rate of a thermodynamically feasible chemical reaction. Therefore, they cannot perform reactions that are not thermodynamically possible. Catalysts reduce the activation energy by increasing a reaction from another path and increase the reaction speed. The relationship between the activation energy of a chemical reaction and its rate of progress is expressed using a quantitative relationship called the Arrhenius equation. According to this relationship, the activation energy can be calculated. The speed constant depends on the temperature. In other words, the reaction rate changes with temperature. The Arrhenius relation shows this dependence.

(5) $$Lnk=\frac{-E_{a}}{RT}+LnA$$

In this equation, A is the collision frequency constant, Ea is the activation energy, R is the gas constant (8.314 joules per mol Kelvin) and T is the temperature in Kelvin. By examining the reaction rate, calculating K at different temperatures, and plotting the above diagram, the values of Ea activation energy and A constant collision frequency can be obtained by using the slope and width from the origin. According to this relationship, when the temperature increases, the kinetic energy increases, so more molecules can overcome the minimum energy. Considering that in this study, silver ions are used as catalysts in the degradation of phoxim, the following results were obtained by investigating the kinetics of phoxim degradation at different temperatures Table 1:

**Table 1:** Second-order reaction degradation rate constant and correlation coefficient of phoxim: Ag ^+^ (1:1 mol ratio) at different temperatures.

Temperature ( °C)	Kinetic equation	k (L/mol^-1^min^-1^)	$A_{0}$	t_1/2_ (min)	R^2^
−18	$\frac{1}{A_{t}}=0.0348t+0.5923$	0.0348	1.7	17.0	0.9987
4	$\frac{1}{A_{t}}=0.0685t+0.5988$	0.0685	1.8	8.7	0.9989
22	$\frac{1}{A_{t}}=0.1161t+0.6382$	0.1161	1.6	5.5	0.9981
25	$\frac{1}{A_{t}}=0.1279t+0.6704$	0.1279	1.5	5.2	0.9985

As you can see, an increase in temperature (higher value of T) gives a greater value of ln k and therefore a higher value of k and since the rate of the reaction depends on the rate constant, k, an increase in k also means an increased rate of reaction.

By drawing Ln k with respect to $\frac{1}{T}$, the following figure will be obtained.

According to the diagram above the slope of their line, the amount of active activation energy can be extracted, which is obtained from the constant global multiplication of gases (R) in the expression $\frac{R}{E_{a}}$. The collision factor is also obtained from the width value from the graph's origin. Using the slope of the energy equation, the activation of phoxim degradation in the presence of silver ions will be 22.6 kJ mol^-1^.

**Fig. 3:**
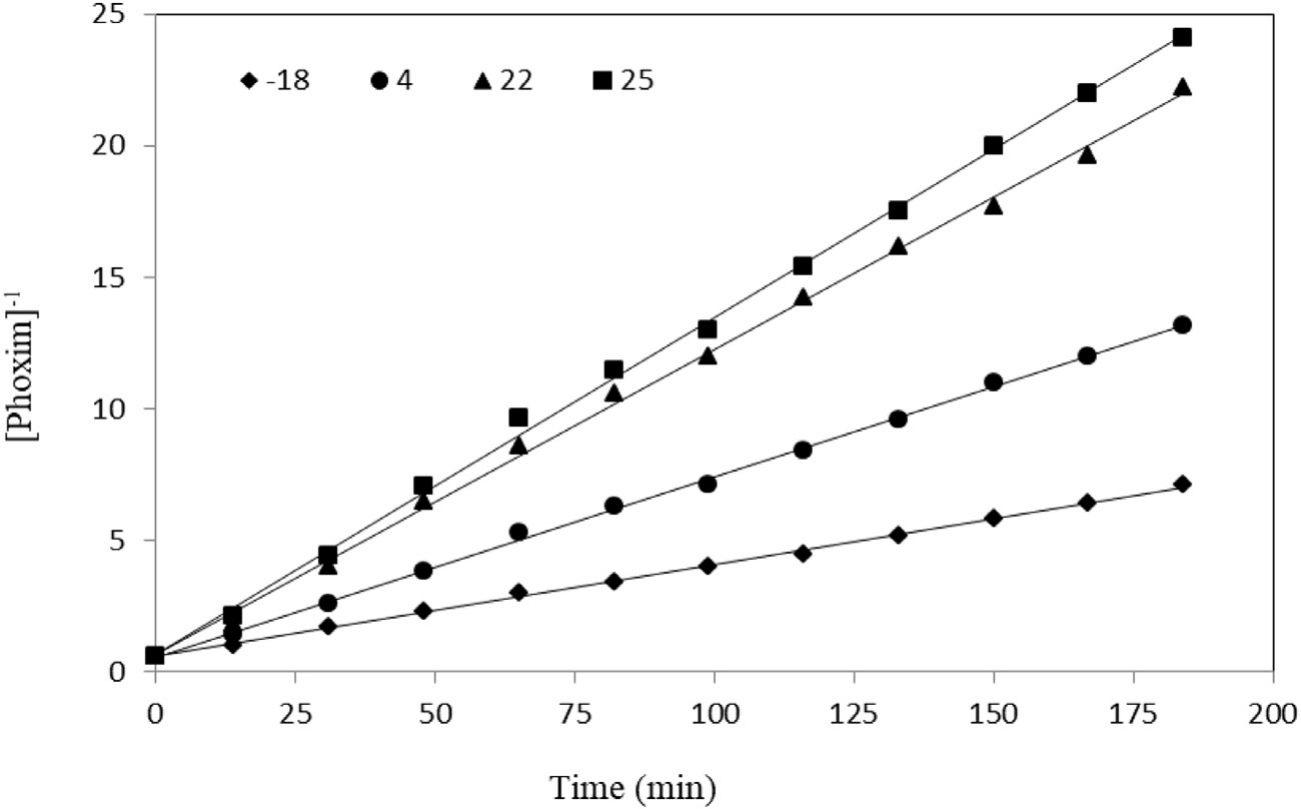
Plot of $\frac{1}{\left[phoxim\right]}$ respect to time for phoxim at different temperatures.

**Fig. 4:**
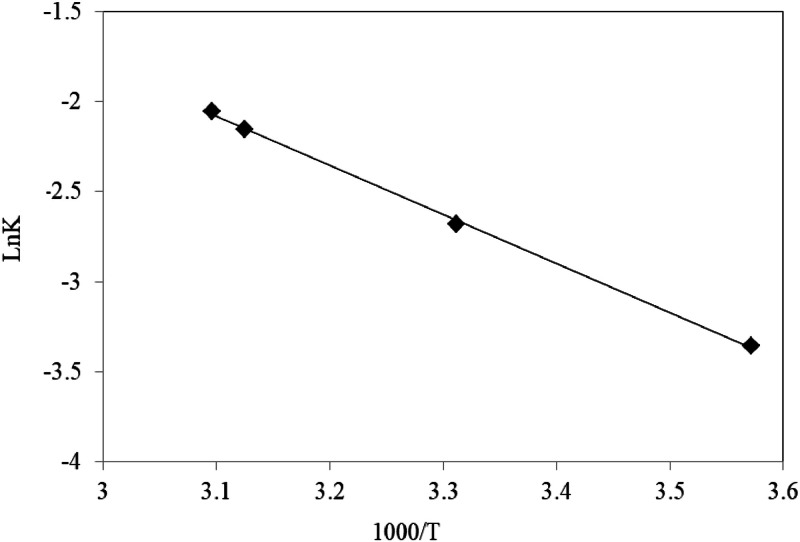
Plot of Ln k respect to $\frac{1}{T}$ for phoxim.

**Fig. 5:**
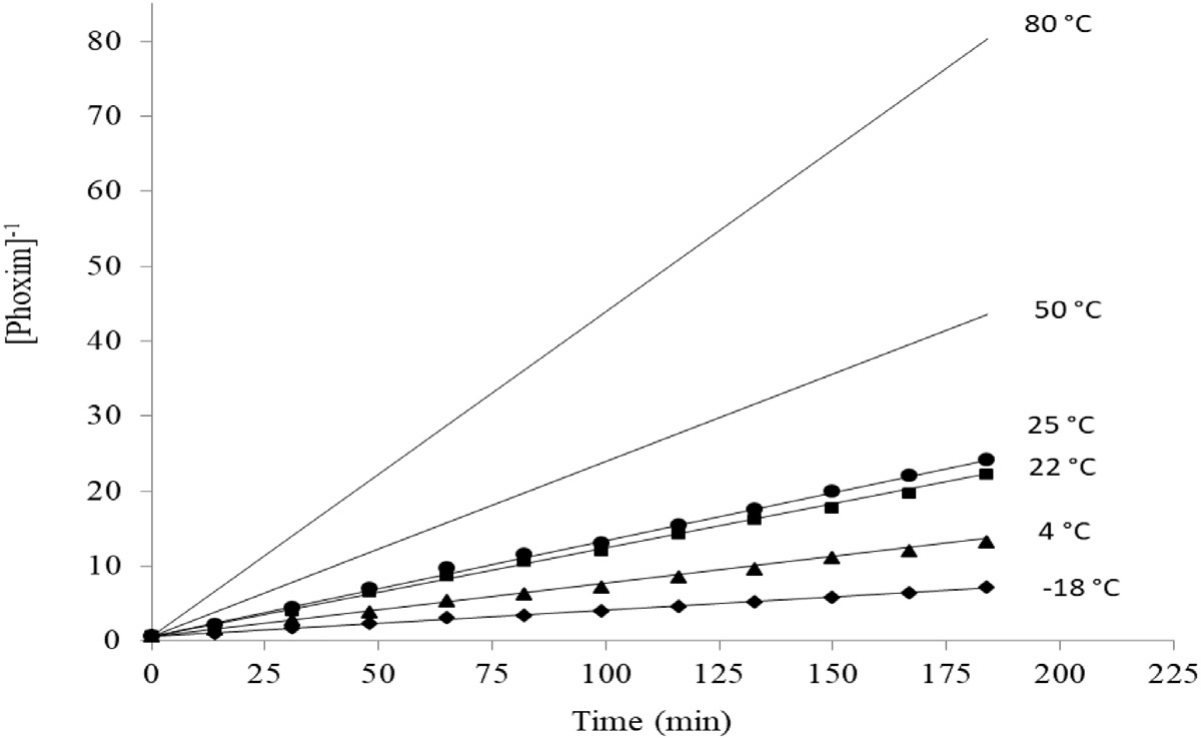
Comparison of experimental (point) and calculated (line) for degradation of phoxim at various temperatures.

Also, we can predicate degradation of phoxim in each temperature by using Arrhenius relation: In the first step, ln k values were plotted against $\frac{1}{T}$ (Fig. 4), and the values were fitted with a straight line by least-squares regression to estimate the Ea and LnA parameters. According to Eq. (1), the plots are expected to be straight lines of similar slopes (correlation coefficients, r2, 0.9991). Finally, these values were used to predict degradation from Eq. (3). Fig. 3 compares the calculated isotherms with the experimental data. As seen, the agreement is satisfactory Fig. 5.

(6) $$k_{i}=\exp\left[\frac{-E_{a}}{RT}+LnA\right]$$

$k_{i}$was obtained in each temperature by using following equation:

(7) $$k_{i}=\exp\left[\frac{-2.7281}{T}+6.3747\right]$$

(8) $$\frac{1}{A_{t}}=k_{i}t+0.6249$$

OPPs whose use-by date has passed and their effective substance has been partially destroyed and its percentage has decreased, or additional OPPs that have been purchased in excess of consumption, must be destroyed. The cost of destroying and destroying and storing these compounds is very high and considering that the methods used to destroy and destroy these compounds, in addition to the high cost, also cause environmental pollution. Using an effective, low-risk, cheap and fast method to destroy them is one of the goals that are being studied and studied by governments and environmental protection organizations today. In cases where we need to degraded the organophosphate pesticide, we can use this technique.

## Conclusion

The results of this study showed that the degradation kinetics of phoxim is second order and two molecules of phoxim react with silver ions and eventually degrade. The rate constant increases with increasing temperature and during 50 min more than 90 % of phoxim destroyed at ambient temperature and the half-life of phoxim at ambient temperature is about 5 min.

## Limitations

Not applicable.

## Ethics statement

This study, titled “The study of kinetic of silver catalytic degradation of phoxim” was conducted in strict accordance with the ethical guidelines of the MethodsX Journal.

## CRediT author statement

Jalal Hassan: Methodology—Analysis; Gholamreza Shams: Data acquisition—Writing; Mohammad Javad Chaichi: Methodology — Supervision; Ali Pourshaban-Shahrestani: Data acquisition—Review and Editing.

## Declaration of competing interest

The authors declare that they have no known competing financial interests or personal relationships that could have appeared to influence the work reported in this paper.

## Data Availability

Data will be made available on request.
